# Human hippocampal pre-activation predicts behavior

**DOI:** 10.1038/s41598-017-06477-5

**Published:** 2017-07-20

**Authors:** Anna Jafarpour, Vitoria Piai, Jack J. Lin, Robert T. Knight

**Affiliations:** 1 Helen Wills Neuroscience Institute, University of California, Berkeley, California USA; 2 Department of Psychology, University of California, Berkeley, California USA; 3 Radboud University, Donders Centre for Cognition, Nijmegen, The Netherlands; 4 Radboud University Medical Center, Department of Medical Psychology, Nijmegen, The Netherlands; 5 Comprehensive Epilepsy Program, Department of Neurology, University of California, Irvine, California USA

## Abstract

The response to an upcoming salient event is accelerated when the event is expected given the preceding events – i.e. a temporal context effect. For example, naming a picture following a strongly constraining temporal context is faster than naming a picture after a weakly constraining temporal context. We used sentences as naturalistic stimuli to manipulate expectations on upcoming pictures without prior training. Here, using intracranial recordings from the human hippocampus we found more power in the high-frequency band prior to high-expected pictures than weakly expected ones. We applied pattern similarity analysis on the temporal pattern of hippocampal high-frequency band activity in single hippocampal contacts. We found that greater similarity in the pattern of hippocampal field potentials between pre-picture interval and expected picture interval in the high-frequency band predicted picture-naming latencies. Additional pattern similarity analysis indicated that the hippocampal representations follow a semantic map. The results suggest that hippocampal pre-activation of expected stimuli is a facilitating mechanism underlying the powerful contextual behavioral effect.

## Introduction

People use prior knowledge to flexibly adapt and speed behavior. This behavioral facilitation is called the temporal context effect and is shaped by expectations built on the flow of preceding events^1, 2^. Contextual memory effects are dependent on the hippocampal complex^3–5^. The hippocampus is a key hub for retrieving associative memories^4, 6–8^ and assisting semantic search^9, 10^. Theoretical models derived from human^2, 11^ and animal data^12^ suggest that the temporal context effect is based on hippocampal pre-activation of expected upcoming stimuli (i.e. pre-play). The representation of predictable sequences has been decoded using functional MRI from the human hippocampus, suggesting that the temporal context probes re-activation of associated stimuli in the sequence^3, 13^. However, the slow time course of fMRI does not allow disentangling the pre-activation to the re-activation of the expected stimulus; thus, a link between pre-activation of hippocampal representations to behavioral facilitation is missing. Using intracranial electroencephalogram (iEEG) recordings from the human hippocampus, we investigated the mechanism underlying the temporal context effect.

**Figure 1:**
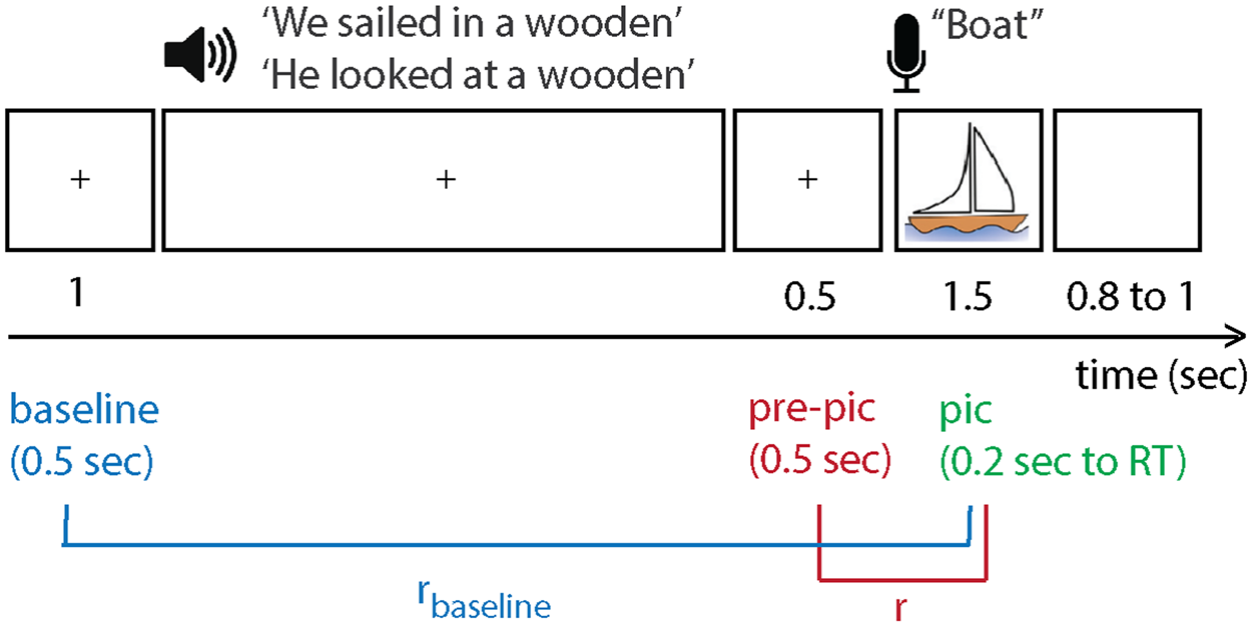
Experimental trial: participants listened to sentences that either built high or low expectations on upcoming pictures (pic) to be named. After a 0.5 s silent gap, participants named the picture that completed the sentence. We tested if the similarity-distance between the pattern of activity in the high frequency band during picture and pre-picture intervals predicted the RTs, by calculating the Spearman correlation coefficient (r). We calculated r_baseline_ as a baseline for the correlation. The pre-activation index was defined as (r−r_baseline_)/r_baseline_. The image of the boat was obtained from http://www.clipartbest.com/clipart-LcK54yLca with CC BY 3.0 license (https://creativecommons.org/licenses/by/3.0/).

We took advantage of sentences that provide a strong temporal context and have shown a robust behavioral facilitation effect^5^. A temporal context is provided by words with semantic associations organized by syntax (a temporal order)^5^. In each trial, participants (n = 5; Table 1) performed a visual-auditory experiment. They listened to a sentence that lacked the final word. After a silent gap (0.5 s) a picture was displayed. There were no visual stimuli prior to the picture onset. Subjects named the picture that completed the sentence (Fig. 1). In half of the trials, the sentences induced high expectations of the upcoming stimuli (high-expected condition), whereas in the other half of the trials they did not (low-expected condition). We observed a behavioral context effect: all participants named the high-expected pictures faster than other pictures (all *P*-values < 0.05; Table 2), in line with previous research^5, 14, 15^. Here we tested if the response times (RT) could be predicted by hippocampal pre-activation of expected stimuli.

**Table 1:** Patients’ information, hemisphere included in the analyses, number of contacts in the hippocampus, and their number and locations. Note: TLE = temporal lobe epilepsy.

Patient	Dominant - Hand	Diagnosis	Coverage	Hemisphere Analyzed	Number of Contacts	Contact Number
1	right	right TLE	bilateral	Left	2	1–2
2	right	right TLE	left	Left	2	3–4
3	right	right frontal encephalomalacia	right	right	2	5–6
4	right	right TLE	bilateral	left	4	7–10
5	right	unidentified seizure focus	bilateral	left	2	11–12

**Table 2:** Patients’ mean response times (in seconds) and the standard deviations in parentheses for each temporal context condition (high or low expectation) and the statistical difference between the two conditions indicated by two-sided Wilcoxon rank sum tests.

Patient	RT: High (S)	RT: Low (S)	P-Value	Z-Value	Rank-Sum (1.0E + 03 X)
1	0.6996 (0.0263)	0.8621 (0.0267)	<0.001	−4.5613	1.4295
2	0.7504 (0.0309)	0.8647 (0.0272)	0.003	−2.9646	1.0440
3	0.7749 (0.0341)	0.9821 (0.0340)	<0.001	−3.7183	1.4955
4	0.9420 (0.0340)	1.0590 (0.0323)	0.010	−2.5665	1.5495
5	0.8282 (0.0324)	0.9597 (0.0297)	0.004	−2.8528	1.2365

## Results

**Figure 2:**
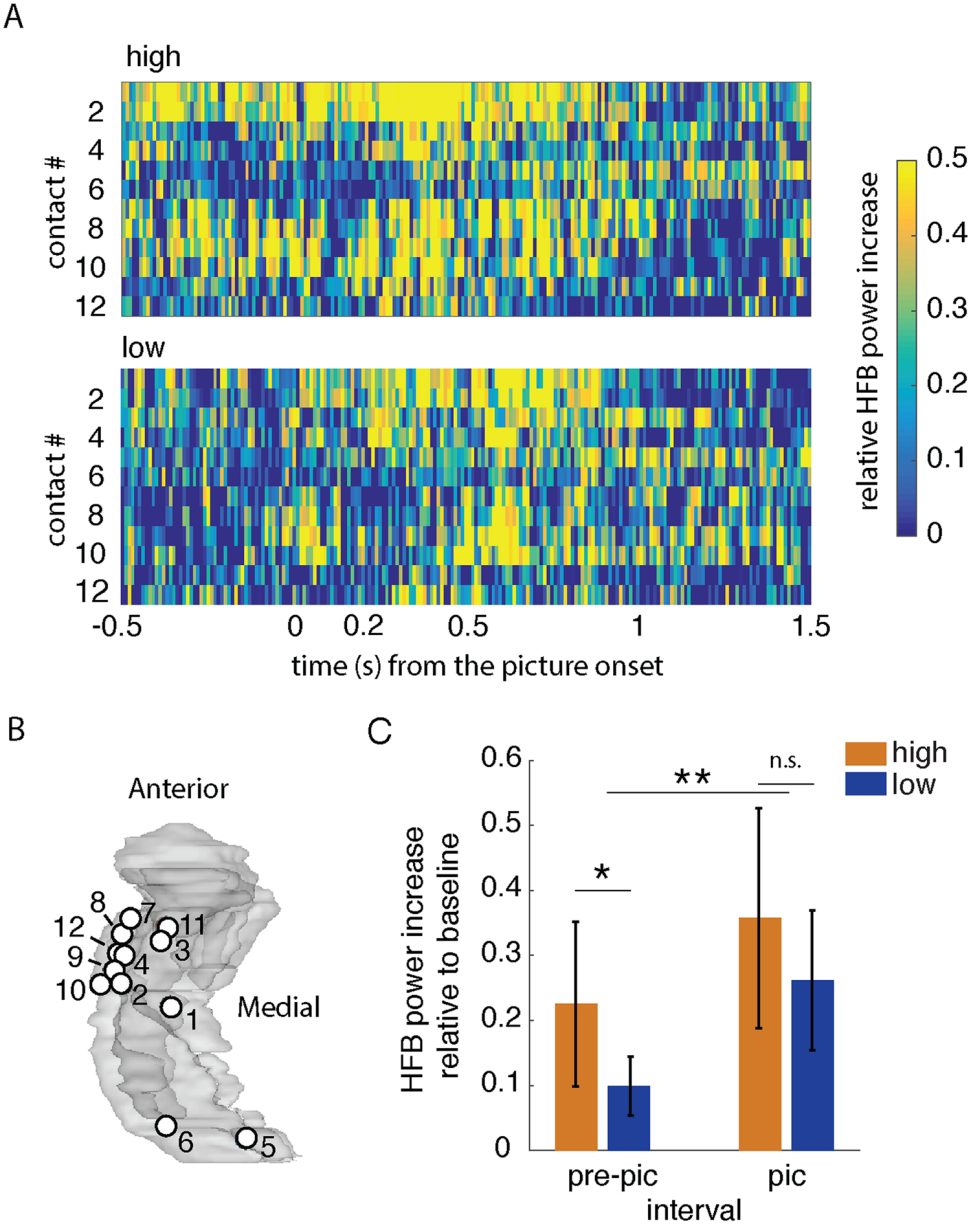
HFB power during pre-picture and picture intervals. (**A**) Averaged HFB power in −0.5 s to 1.5 s from the picture onset (irrespective of the RT) for the high-expected condition (top panel) and the low-expected condition (bottom panel). The HFB power in each trial was relative to the baseline (0.5 s before sentence onset). Each row shows the HFB power in a contact. X-axis is time from onset of the picture. Y-axis is the contact number (see Table 1). (**B**) Map of contacts corresponding to (**A**) in a glass hippocampus. Contacts 5 and 6 were located in the right hippocampus, but are shown in the left glass hippocampus for illustration. (**C**) Average HFB power increase relative to the baseline during the pre-picture interval (0.5 s gap between the offset of the last word from the sentence and the picture onset) and picture interval (0.2 s from onset of the stimuli to RT). The HFB power during the picture interval was more than the power during the pre-picture interval (**P < 0.001). During the pre-picture interval HFB power was more in the high-expected condition than in the low-expected condition (*P < 0.05). The error-bars show the standard deviations and n.s. denotes not significant.

Single-unit activity^16^ and local field potential (LFP) in the high frequency band (HFB, 50–250 Hz)^17, 18^ indicate that picture concepts are represented in the hippocampus approximately 0.2 s after picture onsets (Fig. 2A). Accordingly, we studied the HFB power relative to the baseline (the 0.5 s interval prior to the sentence onset) in the contacts located in the hippocampus (Fig. 2A). Analyses were done separately for each hippocampal contact. We focused on the HFB activity at 0.2 s from the picture onset to the RT for the picture intervals. Congruent with previous reports^17, 18^, we found more HFB power during the picture interval than during the pre-picture interval (0.5 sec gap between the sentence and the picture onset; Wilcoxon signed rank test: *P* < 0.001, 95% CI = (0.102, 0.197), *r* ^*2*^ = 0.797; Fig. 2C). There was no difference between HFB power in strongly expected versus weakly expected trials during the picture interval (Wilcoxon signed rank test *P* = 0.2189, 95% CI = (−0.027, 0.213), *r* ^*2*^ = 0.524). However, there was more HFB power during the pre-picture interval when the upcoming picture was strongly expected than when the picture was weakly expected (Wilcoxon signed rank test *P* = 0.014, 95% CI = (0.0204, 0.2411), *r* ^*2*^ = 0.139).

We tested if the similarity between the pattern of hippocampal LFP power in HFB during the pre-picture intervals and the picture intervals predicted the RTs for high-expected stimuli. For this analysis, the HFB power signal was z-normalized to avoid biases based on power differences between conditions. The similarity metric was calculated using a dynamic time warping algorithm (DTW)^19^. DTW expands or compresses two time-series to find their optimal alignment in time, then measures the ‘similarity-distance’ between the aligned signals. Note that shorter similarity-distance reflects more similar patterns. This pattern similarity analysis was conducted in the temporal domain, separately for each contact.

The pre-activation hypothesis states that the RTs to expected stimuli decreases with the decreasing similarity-distance between neural activity during pre-picture and picture intervals. We tested this by calculating the Spearman’s rank correlation coefficient between RTs and pre-picture to picture similarity-distance in each hippocampal contact (‘r’; Fig. 1). As a control, we also quantified the fit between RTs and the similarity-distance between the baseline (0.5 s pre-sentence onset) and the picture interval (r_baseline_; Fig. 1). The pre-activation index was then formulated as: (r−r_baseline_)/r_baseline_. A positive pre-activation index indicates that the distance between pre-picture and picture intervals better predicted RTs than the distance between baseline and picture intervals. The pre-activation index was higher for high-expected (mean = 0.1564, SD = 0.1912) than low- expected pictures (mean = −0.0664, SD = 0.1974; linear mixed-effects model considering the uneven contribution of contacts across patients: parameter estimate = −0.0025; t(22) = −2.9343, *P* = 0.008, 95% CI = (−0.380, −0.065)).

**Figure 3:**
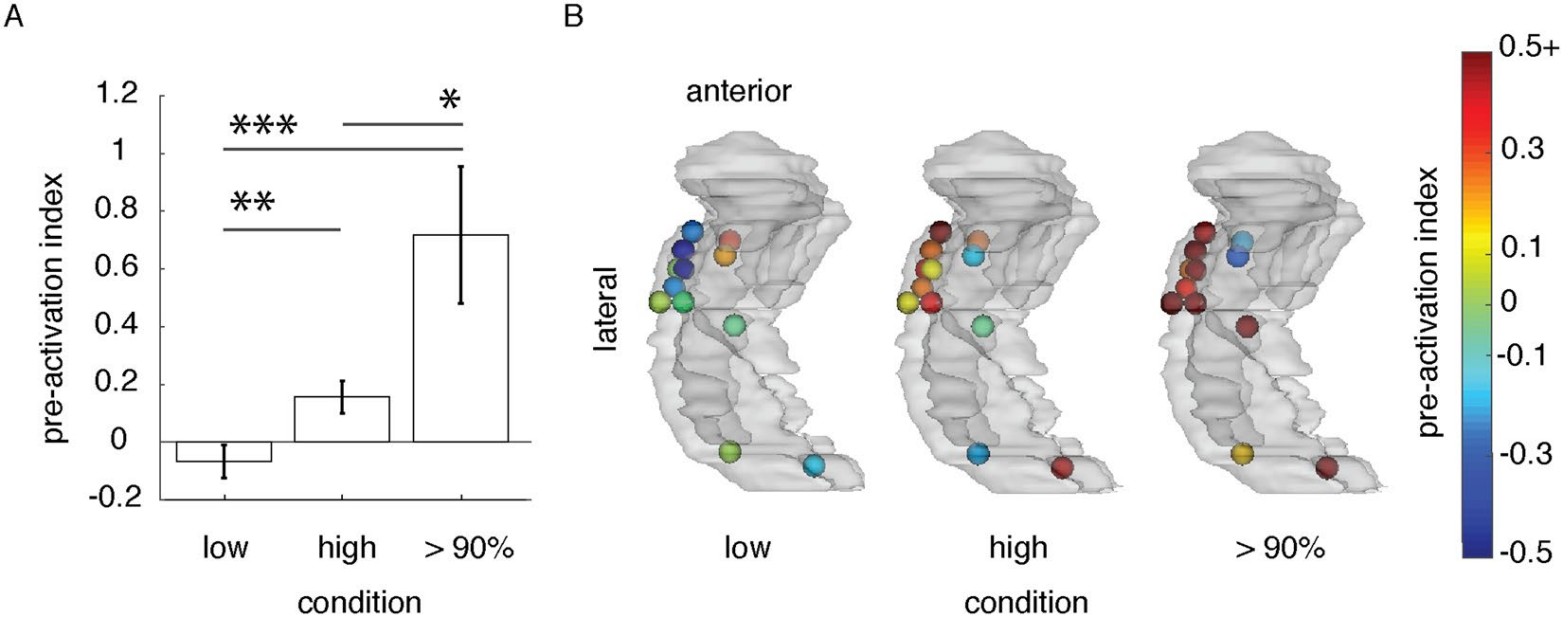
Pre-activation indexes (**A**) for the high-expected and low-expected trials, and a subset of high-expected trials for which the picture stimuli were predictable with more than 90% certainty. The error-bars show the standard error of the mean. *P < 0.05, **P < 0.01, *** = *P* < 0.001. (**B**) a left glass-hippocampus shows the topography of contacts and the values of pre-activation index as shown in (A). Contacts are color-coded by the pre-activation index value which ranged between −0.5 to 0.5 + (the pre-activation indices above 0.5 are color-coded in dark red).

We followed this result further with a post-hoc analysis. We focused on the subset of trials in the high-expected condition for which the upcoming picture was expected with more than 90% certainty. An independent study measured the certainty by asking subjects to complete the sentences with the word of their choice (no picture naming). The proportion of subjects who completed a sentence with the same word was calculated to yield the certainty of the expected word. For example, a picture-name was expected with more than 90% certainty if more than 90% of subjects used the same word for completing a sentence. We observed that the pre-activation index was higher for the trials with more than 90% certainty on expected stimuli than for low-expected stimuli (mean = 0.7173, SD = 0.8212, Fig. 3; parameter estimate = −0.80; t(22) = −4.0148, *P* < 0.001, 95% CI = (−1.21, −0.387)). The results are in agreement with the hypothesis that the pre-activation of expected stimuli facilitates behavior.

We observed a correlation between pre-activation and RT. To assess how specific the pre-activated hippocampal pattern was to the picture a post-hoc analysis was done on 9 out of 12 contacts. These results supported the pre-activation hypothesis when the probability of expected stimuli was more than 90% (the red contacts in Fig. 3B). The trials with high-expected pictures for which RT was longer than the median RT in the low-expected condition were excluded. In those trials, we assumed that the participants did not benefit from the strong context in the sentence, which is reflected in slow RT responses. We observed that the similarity-distance between pre-picture and picture intervals in the high-expected trials (mean = 0.365, SD = 0.019) was smaller than the similarity-distance in the low-expected trials (mean = 0.388, SD = 0.027, Fig. 3; parameter estimate = 0.023; t(16) = 2.197, *P* = 0.043, 95% CI = (0.001, 0.044)). In other words, the pattern of HFB power during pre-picture and the picture intervals were more similar to each other in high-expected trials than in low-expected trials.

**Figure 4:**
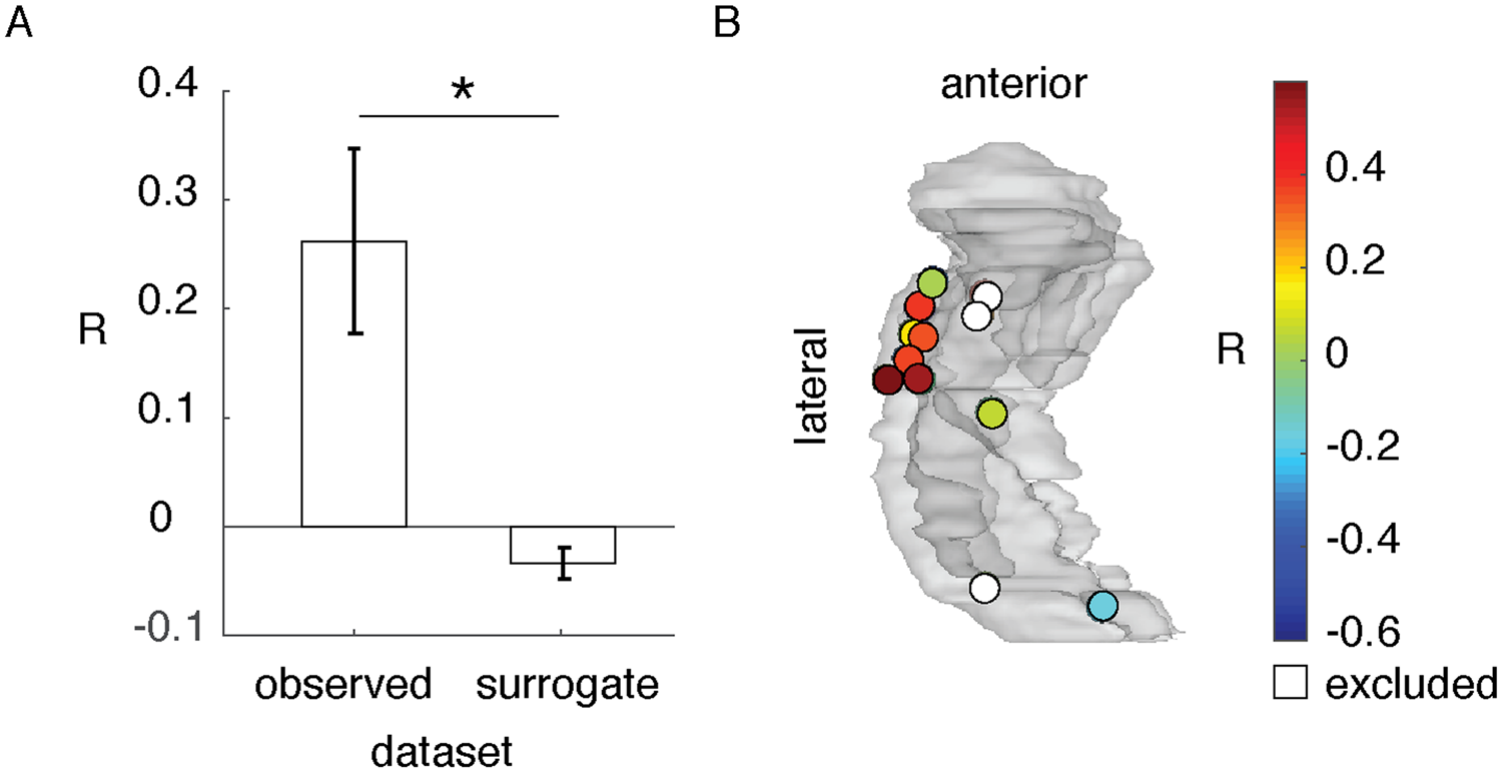
Similarity-distance and semantic distance correlation coefficient (R) **(A)** comparing the data with a surrogate permutation shows that the similarity-distance between HFB pattern during the pre-picture interval of an expected picture and other picture intervals correlated with the semantic distance of the expected picture concept and the other picture concepts (*P < 0.05). **(B)** shows the value and topography of observed R (left bar in **A**) in a glass hippocampus (see Fig. 2B). The white contacts were excluded from this post-hoc analysis.

To test the specificity of hippocampal representation of the pictures, we compared the similarity-distance between pre-picture interval in trial *i* and picture interval in the same trial, to the pre-picture interval in trial *i* and the picture interval in all other trials *k* = 1 … *n*, *k* ≠ *i*. We did not observe a significant difference between them (parameter estimate = 0.008; t(16) = 1.697, *P* = 0.108, 95% CI = (−0.003, 0.033)). However, we observed that the similarity-distance between the pre-picture interval of trial *i* and the other picture intervals increased with increasing distance in semantic association (from latent semantic analysis) of the expected picture concept *i* and the other picture concepts (mean correlation coefficients = 0.262, SD = 0.255) relative to the surrogate data. The surrogate data was built by random permutations of other trials index *k* in the array of semantic association distance (mean correlation coefficient = −0.025, SD = 0.069; parameter estimate = 0.083; t(16) = −3.46, *P* = 0.003, 95% CI = (−0.46, −0.11); Fig. 4). For example, the pattern of activity prior to the picture of a ‘boat’ was more similar to the picture of a ‘map’ than to the picture of a ‘key’, because ‘boat’ has closer semantic association to ‘map’ than to ‘key’. Note that in our experimental design the semantic association across picture concepts was small (mean LSA = 0.122, SD = 0.105, LSA range is between 0 and 1). This result suggests that hippocampal activity prior to the expected stimuli contains information about the semantic domain of the upcoming stimuli.

## Discussion

We tested if the hippocampus represents expected stimuli prior to their appearance, and if this pre-activation facilitates behavior. Epileptic patients performed a picture-naming task while we recorded LFP from their hippocampus using iEEG (Table 1). In half of the trials the pictures were high-expected because it was probed by a constraining temporal context; and in other trials the pictures were low-expected because these followed an un-constraining context (Fig. 1). Participants were faster in naming the expected pictures than other pictures (Table 2) – i.e. the temporal context behavioral effect. We found that hippocampal HFB power was enhanced in the high-expected condition in comparison to the low-expected condition, in the gap between the context and the upcoming picture (pre-picture interval; Fig. 2). We also observed that the pattern of hippocampal HFB activity in the pre-picture interval was more similar to the picture-interval when the pictures were high-expected than when they were low-expected. Further, the decreasing similarity-distance between the pre-picture and picture intervals correlated with decreasing RT (Fig. 3). Thus, the responses were faster when the hippocampal representations of expected stimuli were pre-activated. Our results suggest that the hippocampus pre-activation is a mechanism underlying the temporal context effect.

People use their knowledge to facilitate responses to expected upcoming events. In our experiment, the expected stimuli were knowledge based, rather than a fixed experimental sequence of unrelated items. Previously, using fMRI, an experimentally expected stimulus is decodable from the hippocampus given a predictive sequence of items^13^ or temporal context^3^. Hsieh *et al*. (2014) decoded objects in a sequence when the objects were in the expected sequential position, rather than when they were in an unexpected position^3^. In rodents, the hippocampus pre-activates expected stimuli already prior to the onset of a task^12, 20^. This ‘pre-play’ was observed only when a reward was expected at that cued place^12^; the pre-play was followed by navigating toward the reward-expected place.

Here we decoded pre-activations in the human hippocampus from single contacts, based on iEEG. It is difficult to decode pre-activations right before stimuli presentation with fMRI-based pattern similarity analysis, due to the low temporal resolution of fMRI. We decoded the pre-activation of hippocampal representations using the temporal pattern of HFB activity. We applied a dynamic time warping algorithm that has been mainly used for alignment of sound signals^19^ or for decoding the temporal sound profile from the neural activity^21^. Advantages of this decoding approach are that the algorithm does not assume a speed on pre-activation (how much faster or slower the pre-activation is than the stimuli representation), and it is based on the LFP in a single contact, making the results comparable to the hippocampal multi-unit or single unit neural activity^22, 23^.

Single unit recording from the hippocampus suggests that the hippocampal representations of pictures are conceptual, because the same neuron is active for the picture, the written name, or the sound of a concept^16, 24^. Here we found that the pre-activation of hippocampal picture presentation was related to the semantic relationship across pictures. The similarity between a pre-picture interval to all other pictures correlated with the semantic similarity between the picture concept with the other picture concepts (Fig. 4). This result is in agreement with reports of hippocampus engagement in establishing and accessing semantic memories^25–27^. Previous work on the human hippocampal LFP showed that the event-related response is modulated by semantic priming^28, 29^: the hippocampal event-related response to a word (e.g. pepper) is reduced following the response to a semantically related word (e.g. salt). Hence, the hippocampal representations of words may overlap in their semantic fields.

Our result suggests that in addition to the conceptual representation of pictures^24^, the hippocampus may contain a semantic map of conceptual relations, akin to the hippocampal spatial map. Spatial representation in the hippocampus was first reported in rodents^30^. Recent evidence from rodents suggests that the hippocampus represents both spatial and non-spatial stimuli^31^. In humans, the hippocampus represents space^32^ and the spatial relation of places (e.g. the Euclidian distances between places)^33^. The hippocampi of experts with extensive training on associated stimuli (e.g., taxi-drivers with extensive knowledge of spatial associations^34^ and musical experts with extensive knowledge about complex sound associations^35^) are bigger than the hippocampus of non-experts, supporting the hypothesis that the hippocampus represents maps of associations. In line with this, the hippocampus may also reflect semantic associations. Future studies are required to examine in detail the semantic representation of concepts in the hippocampus.

In this study, we decoded the hippocampal pre-activation of expected stimuli prior to their presence. The decoding was based on the pattern of HFB activity in the hippocampus, acquired by iEEG. We showed that pre-activations facilitated responses to expected stimuli. The RT was shorter when the representation of an expected stimulus emerged prior to its onset. Our findings provide the first electrophysiological evidence in humans linking a contribution of hippocampal pre-activation in shaping behavioral responses to expected stimuli, such as that observed in language production^5, 9, 27^.

## Methods

### Approval

The study protocol was approved by the Office for the Protection of Human Subjects of the University of California, Berkeley, the University of California, Irvine, and Stanford University. All patients provided written informed consent before participating and all methods were performed in accordance with the relevant guidelines and regulations.

### Participants

Five participants (two females, mean age = 42, sd = 9.3, range = 32–52) were implanted stereotactically with depth electrodes to localize the seizure onset zone for subsequent surgical resection (Table 1). The electrodes were placed at the University of California, Irvine Medical Centre (4 patients) or at Stanford University, Medical Centre (1 patient) with 5-mm inter-electrode spacing. All participants had normal hearing and normal vision. No seizures occurred during task administration. Only contacts in the hippocampal complex of the non-epileptic brain area were included for analysis (Table 1). These contacts were inspected by two independent neurologists to confirm absence of interictal epileptiform activity.

### Materials

Participants performed a multimodal experiment. They were instructed to listen to incomplete sentences and name the picture that appeared on a computer screen after a silent gap. (The stimuli were acquired from http://freevectordownloadz.com/). Sentence semantic associations and sentence syntax (temporal order of words) provided a temporal context effect. Each picture (n = 51) was named twice: once proceeding a sentence building a high expectation for the upcoming picture, and once with a low expectation (Fig. 2). All sentences (N = 102) had six syllables. The sentences were spoken by a female native speaker of American English at a regular pace of 2.11 syllables per second. The mean duration of the sentences was equal across conditions (*t*(50) < 1). We used auditory stimuli for the context for two main reasons. First, auditory presentation mitigates the issue of individual differences in reading speed that would have been if sentences were presented visually. Accordingly, all participants encountered the context and pictures with the same pace. Second, multimodal representation of concepts has been found in the hippocampus^16^, which is the focus of this research.

In half of the trials, sentences built weak expectation (many concepts would complete the sentence); in the other half of the trials, the temporal context built a high expectation for the upcoming picture. For example, “We sailed in a wooden” has a high expectation for ‘boat’. In contrast, “He looked at a wooden” has a low expectation for ‘boat’, because any visible wooden concept would complete the sentence (Fig. 2).

A separate pre-test experiment confirmed that sentences in the two conditions differed in the degree of expectancy on the final word, *t*(50) = 45.928, *p* < 0.001. Fourteen volunteers completed the sentences and expectation probability was calculated as the proportion of volunteers who completed the sentence with the target picture-name. Our target words had a mean completion probability of 83% for the high expectation condition and 4% for low expectation condition.

### Procedure

Stimulus presentation was controlled by Presentation (Neurobehavioral Systems, Albany, CA). Participants were instructed to listen to the sentences and name the pictures as soon as they saw the picture on the screen. The sentences were presented via stereo loudspeakers. A trial began with a white fixation cross on a black background, displayed continuously during sentence presentation. After 1 s, the sentence was presented. The fixation cross remained on the screen after the sentence offset for another 0.5 s. After this silent gap, the picture was displayed for 1.5 s, when participants responded. A black screen was then presented for an interval varying between 0.8 and 1.5 s (Fig. 2). A unique randomized list of materials was used for each participant^5^.

### Behavioural Analysis

Trials with disfluent responses, omissions, unrelated responses, or responses longer than 1.5 s were excluded from all analyses. Vocal responses were recorded with a microphone via Presentation and manually analysed using Praat^36^ for the detection of speech onset (the experimenter was blind to the condition label of each trial). A two-sided Wilcoxon rank sum test was used to compare response time (RT) in the two conditions for each patient separately (Table 2).

### EEG data collection and pre-processing

Intracranial EEG data were acquired using the Nihon Khoden recording system, analog-filtered above 0.01 Hz and digitally sampled at 5 KHz or 10 KHz. The loudspeakers and a photodiode were recorded as analogue channels to mark the beginning of the sentences and the presentation of the picture respectively. A neurologist selected the contacts that were in the hippocampus of the non-epileptic hemisphere for inclusion in the analyses. Twelve contacts were included in the analysis. Four patients had two hippocampal contacts and one patient had four contacts (Table 1, Fig. 2B).

All EEG analyses were run in Matlab 2014a, EEGLAB^37^, and Fieldtrip^38^ offline. We applied a 2 Hz stopband Butterworth notch filter at 60 Hz line power noise and harmonics. The EEG was de-trended (i.e., the mean value of the entire signal at each contact was subtracted for the value at each time point), high-pass filtered at 0.5 Hz using a zero-phase delay finite impulse response filter with Hamming window (*fir1* in Matlab), and then down-sampled to 1 Khz using Matlab’s resample() function. All contacts were then re-referenced to the nearest white-matter contact.

Artifact rejection was performed over raw data segments for the baseline period (−0.5 s pre-sentence onset to sentence onset) and between 1 s before and 2 s after picture onset. First, using the raw signal, any trial segment with a data point exceeding 5.8 standard deviations (SD) from the mean was excluded^5^. To examine fast changes in the signal, adjacent time points were subtracted in a sequential fashion, and trial segments with any points exceeding 8 SD were excluded. These thresholds were chosen to remove segments with outlier data points while keeping as many trials as possible^5^. On average across participants, there were 39.4 (SD = 9.2) trial in high expectation and 41.40 (SD = 6.27) low expectation (*P* > 0.05).

Finally, the signal was bandpass filtered at 50 to 250 Hz with a Hamming window. The Hilbert transformation was applied on the filtered data to extract the power in the high-frequency band (HFB) between 50 and 250 Hz. This signal was later used for HFB-power intensity analysis and HFB-power pattern-similarity analysis.

### Pattern similarity analysis

Patterns of HFB power were studied irrespective of the power intensity. The epochs of interest were:

- The picture interval: 0.2 s post-picture onset to RT
- The pre-picture interval: 0.5 s before picture onset. We included the entire 0.5 s pre-picture interval in the analysis because we did not have a prior assumption for when and how fast there would be a stimulus pre-play
- The baseline interval: 0.5 s pre-sentence onset (same length as the pre-picture interval).

The HFB (50 to 250 Hz) power in these epochs (picture interval, pre-picture interval, and baseline interval) were down-sampled to 100 Hz for computational purposes^21^ and then each epoch was z-normalized in the time dimension. Dynamic time warping (DTW)^19, 21^ was applied to measure the distance between patterns of HFB power before and during the picture interval. DTW expands or compresses two time-series to find their optimal alignment in time. Initially, this algorithm calculates the minimum Manhattan distance (the absolute value of the difference) between each time point during the picture interval (i = 1 to n; n is the length of the picture interval) and all time points during the other interval (baseline or pre-picture interval: j = 1 to m; m is the signal length). This generates a cost matrix, M (n by m). The distance between two warped signals is defined by the lowest cost path from the beginning of the signals, M(i = 1, j = 1), to the end, M(i = n, j = m). To navigate in the cost matrix from point (1, 1) to (n, m) one of the following steps is allowed: expansion (moving to (i + 1, j)), compression (moving to (i, j + 1)), or matching (moving to (i + 1, j + 1)). The steps are chosen to find the lowest cost path, which would be the *distance* between two warped signals.

The algorithm allowed us to measure the distance between temporal profiles of two time-series with different lengths. DTW penalized fast or slow pre-plays by quantifying a large distance. Two unrelated sequences also have a large distance. Distance increases with increasing difference between the lengths of two signals. Therefore, we normalized the distance by the signal length.

We tested the hypothesis of how good distance predicted RT (i.e., fit), and how much that fit was exclusive to the pre-picture interval. For each trial, we extracted the fit (Spearman’s ranked correction coefficient, r) of RT to distance between activity in the picture interval and pre-picture interval. To study its exclusivity, we computed the fit of RT to distance between the picture interval and the baseline (r_baseline_; Fig. 1). The relative difference between the two correlation coefficients was used as the pre-activation index. Thus, the pre-activation index was formulated as (r−r_baseline_)/r_baseline_. An index above zero would mean that the RT could be predicted from the pattern similarity between the pre-picture and picture intervals. The pre-activation index was measured separately for high and low expectation trials, and again for trials for which the upcoming picture was expected with more than 90% certainty.

### Statistical testing

We used a two-sided Wilcoxon rank sum test, in R^39^, across participants to compare the HFB power. However, patients contributed more than one contact to the analyses and, as such, the observations at each contact were not independent. To adjust for this issue, we fitted a linear mixed-effects model to compare the value of the pattern similarity analysis between the two conditions (as a fixed effect: low versus high), with patients as a random effect (Matlab R2016a, statistics and machine learning toolbox, fitlme() function; for example: “decoding_index ~ condition + (1|patient))”. In this way, different contacts are examined as belonging to their respective patient, thus taking the issue of dependence of observations into account. Similar linear mixed-effects models were applied for the statistical comparisons in all post-hoc analysis.

### Post-hoc tests

We compared the similarity-distance between pre-picture and picture intervals in high expected (more than 90% certainty) and low expected trials. The contacts which supported the hypothesis were included (9 out of 12 contacts, shown in red in Fig. 3B). We excluded the trials in the high expected condition for which RT was longer than the median RT in the low expected condition. We assumed that in those trials participants were not engaged in the experiment and, thus, the corresponding data does not show the benefit from the high context.

We also investigated the specificity of the pattern of pre-activation to the upcoming stimuli. The similarity-distance between pre-picture and picture intervals was compared to the distance between pre-picture interval and all other picture intervals (*D*_*ij*_, where *i* is the expected picture, and *j* is a picture in another trial). For example, the similarity-distance between the pre-picture of ‘book’ and picture ‘book’ was compared to the average similarity-distance between the pre-picture of ‘book’ and all other pictures, in each contact.

Next, we tested if the conceptual similarity of the pictured stimuli is reflected in the similarity-distance measurement. For that, we examined the distance in semantic fields. The distance in semantic fields of the pictures was extracted from latent semantic analysis (LSA)^40^: lsa.colorado.edu/. Distance was formulated as 1−*LSA*_*ij*_, where *i* is the high-expected picture, and *j* is a picture in another trial. Accordingly, a larger number means a larger distance in semantic fields. For each contact, we measured the Spearman correlation coefficient between *D*_*ij*_ (similarity-distance between a pre-picture interval and other pictures) and 1−*LSA*_*ij*_ (the distance in semantic field of the picture concepts). We predicted that the measured correlations would be stronger than in the surrogate data. The surrogate data were random selections of pairs of picture concepts. They were repeated 50 times. The averaged correlation coefficients of observed data and the averaged correlation coefficients of surrogate data were compared across the contacts.
